# Co-circulation of Multiple SARS-CoV-2 Variants of Concern in Dhaka, Bangladesh, during the Second Wave of the COVID-19 Pandemic

**DOI:** 10.1128/mra.00549-22

**Published:** 2022-07-11

**Authors:** M. Abdullah Omar Nasif, Nahida Sultana, Raad Rahmat, Tahmina Akther, Afzalun Nessa, Munira Jahan

**Affiliations:** a Department of Virology, Bangabandhu Sheikh Mujib Medical University, Shahbag, Dhaka, Bangladesh; b Biotechnology Program, Department of Mathematics and Natural Sciences, School of Data and Sciences, BRAC University, Mohakhali, Dhaka, Bangladesh; DOE Joint Genome Institute

## Abstract

Thirty partial coding DNA sequences (CDSs) of the spike gene of severe acute respiratory syndrome coronavirus 2 (SARS-CoV-2) were obtained from nasopharyngeal swab samples collected in March to July 2021 in Dhaka, Bangladesh, during the second wave of the coronavirus disease 2019 (COVID-19) pandemic, using Sanger sequencing technology. Sequence analysis showed the presence of multiple WHO-designated variants of concern (VOCs), including Alpha, Beta, Gamma, and Delta, with predominant circulation of Delta variants during that period.

## ANNOUNCEMENT

Severe acute respiratory syndrome coronavirus 2 (SARS-CoV-2), a member of the *Betacoronavirus* genus of the *Coronaviridae* family and the third documented spillover of an animal coronavirus to humans, has caused the devastating worldwide coronavirus disease 2019 (COVID-19) pandemic (1). In Bangladesh, after the first detection on 8 March 2020, the virus caused a relatively small number of cases throughout the year (2). However, there was a rapid surge of cases from March 2021, which reached its peak in July 2021 (2). In this regard, our project aimed to supplement the sequencing efforts in the country and to reveal the variants circulating during the devastating second wave of the COVID-19 pandemic in Bangladesh.

Nasopharyngeal swab samples were collected from patients attending a fever clinic at Bangabandhu Sheikh Mujib Medical University (Dhaka, Bangladesh) in March to July 2021. Real-time reverse transcription (RT)-PCR to detect SARS-CoV-2 RNA was performed using the Sansure Biotech novel coronavirus (2019-nCoV) nucleic acid diagnostic kit. A total of 30 samples with cycle threshold (*C_T_*) values of <25 for both open reading frame 1ab (ORF1ab) and the N gene were selected for sequencing of the spike gene. In short, viral RNA was extracted from the nasopharyngeal swab samples using the PureLink viral RNA/DNA minikit (Invitrogen, USA). First-strand cDNA was synthesized using the Promega GoScript RT system (Promega Corp., USA). Random hexamers were used during this step. Targeted amplification using four sets of primers (3), which covered the whole spike gene and produced 965-, 1,361-, 1,204-, and 1,125-bp overlapping DNA fragments, was performed using GoTaq G2 hot start PCR master mix (Promega Corp.). PCR products were electrophoresed in a 1.5% agarose gel stained with ethidium bromide to determine the DNA fragments of specified length. A 100-bp PLUS Blue DNA ladder (GeneON, Germany) was used as a molecular marker during the electrophoresis process. Amplified PCR products were then purified with the ExoSAP-IT Express PCR product purification kit (Applied Biosystems, USA). Cycle sequencing reactions were performed using the BigDye terminator v3.1 cycle sequencing kit (Applied Biosystems) using both forward and reverse primers (same primer sets as used during the targeted amplification process), followed by purification of the cycle sequencing products with the BigDye XTerminator purification kit (Applied Biosystems). Automated capillary electrophoresis was performed with the purified cycle sequencing products in a 3500 Genetic Analyzer (Applied Biosystems). Sequence trace quality was assessed with Sequence Scanner 2 v2.0 (Thermo Fisher Scientific, USA), and sequences with overall trace scores of >20 were accepted and used for further analysis. Chromatograms were edited in MEGA v11 (4), and four consensus sequences were generated for each sample and then merged using the EMBOSS merger tool (https://www.bioinformatics.nl/cgi-bin/emboss/merger) to generate the spike gene sequence. Sequences were uploaded to the Nextclade web server (https://clades.nextstrain.org) for variant calling, lineage determination, and detection of nucleotide and amino acid mutations in reference to the Wuhan-Hu-1/2019 strain (GenBank accession number MN908947) (5).

In total, 30 partial spike coding DNA sequences (CDSs) were obtained. In variant analysis, 18 sequences (60%) were assigned to Delta, 8 sequences (26.7%) to Beta, 1 sequence (3.3%) to Alpha, and 1 sequence (3.3%) to Gamma. The remaining 2 sequences (6.7%) were assigned to Nextstrain clade 20B. Among Delta variants, Nextstrain clade 21A (Phylogenetic Assignment of Named Global Outbreaks [PANGO] lineage B.1.617.2) was the predominant clade. The sequence identifier, Global Initiative on Sharing All Influenza Data (GISAID) accession number, GenBank accession number, amino acid substitutions, Nextstrain clade, and PANGO lineage for each sequence are listed in Table 1.

**TABLE 1:** Amino acid mutations and phylogenetic traits of sequenced partial spike genes

Sequence identifier	GISAID accession no.	GenBank accession no.	Amino acid substitutions	Nextstrain clade (WHO designation)	PANGO lineage (Nextclade)
BSMMU_Viro_1(258)	EPI_ISL_12845636	ON384009	L18F, D80A, D215G, K417N, E484K, N501Y, D614G, A701V	20H (Beta, V2)	B.1.351
BSMMU_Viro_2(12)	EPI_ISL_12845637	ON384010	L18F, A67V, D80A, D215G, K417N, E484K, N501Y, A522, D614G	20H (Beta, V2)	B.1.351.3
BSMMU_Viro_3(502)	EPI_ISL_12845638	ON384011	Y144F, N501Y, A570D, D614G, P681H, T716I, S982A, D1118H	20I (Alpha, V1)	B.1.1.7
BSMMU_Viro_4(13)	EPI_ISL_12845639	ON384012	L18F, A67V, D80A, D215G, K417N, E484K, N501Y, D614G, A701V, S1252F	20H (Beta, V2)	B.1.351.3
BSMMU_Viro_5(129)	EPI_ISL_12845640	ON384013	L18F, A67V, D80A, D215G, Y313L, Q314F, K417N, E484K, N501Y, D614G, A701V	20H (Beta, V2)	B.1.351.3
BSMMU_Viro_6(160)	EPI_ISL_12845641	ON384014	L18F, A67V, D80A, D215G, K417N, E484K, N501Y, D614G, A701V	20H (Beta, V2)	B.1.351.3
BSMMU_Viro_7(274)	EPI_ISL_12845642	ON384015	L18F, T20N, P26S, D138Y, R190S, K417T, E484K, N501Y, K558R, D614G, H655Y, T1027I, V1176F	20J (Gamma, V3)	P.1.14
BSMMU_Viro_8(42)	EPI_ISL_12845643	ON384016	D80A, W152R, D215G, K417N, E484K, N501Y	20H (Beta, V2)	B.1.351
BSMMU_Viro_9(136)	EPI_ISL_12845644	ON384017	D80A, D215G, K417N, E484K, N501Y, D614G, A701V	20H (Beta, V2)	B.1.351
BSMMU_Viro_11(643)	EPI_ISL_12845645	ON384018	T95I, E484K, D614G, P681H, D796H, S1252F	20B	B.1.1.318
BSMMU_Viro_12(236)	EPI_ISL_12845646	ON384019	T95I, E484K, D614G, P681H, D796H	20B	B.1.1.318
BSMMU_Viro_13(126)	EPI_ISL_12845647	ON384020	L18F, A67V, D80A, D215G, K417N, E484K, N501Y, D614G, A701V, P1263L	20H (Beta, V2)	B.1.351.3
BSMMU_Viro_14(563)	EPI_ISL_12845648	ON384021	T19R, G142D, R246T, L452R, T478K, D614G, P681R, D950N, C1236F	21A (Delta)	B.1.617.2
BSMMU_Viro_16(251)	EPI_ISL_12845649	ON384022	T19R, G142D, L452R, Q474H, T478K, D614G, P681R, D950N	21A (Delta)	B.1.617.2
BSMMU_Viro_17(572)	EPI_ISL_12845650	ON384023	T19R, G142D, Q173H, L452R, T478K, D614G, V615A, P681R, D950N	21A (Delta)	B.1.617.2
BSMMU_Viro_18(59)	EPI_ISL_12845651	ON384024	T19R, G142D, E324Q, L452R, T478K, D614G, P681R, R815T, D950N	21A (Delta)	B.1.617.2
BSMMU_Viro_19(551)	EPI_ISL_12845652	ON384025	Q14R, T19R, G142D, L452R, T478K, D614G, A623S, P681R, D950N, S1252F	21A (Delta)	B.1.617.2
BSMMU_Viro_20(43)	EPI_ISL_12845653	ON384026	T19R, T95I, G142D, L452R, T478K, V608I, D614G, P681R, S943I, D950N, P1263L	21J (Delta)	AY.119
BSMMU_Viro_21(511)	EPI_ISL_12845654	ON384027	T19R, G142D, L452R, T478K	21A (Delta)	B.1.617.2
BSMMU_Viro_22(196)	EPI_ISL_12845655	ON384028	T95I, G142D, L452R, T478K, D614G, P681R, D950N, V1104L, G1251V	21J (Delta)	B.1.617.2
BSMMU_Viro_23(621)	EPI_ISL_12845656	ON384029	G142D, L452R, T478K	21A (Delta)	B.1.617.2
BSMMU_Viro_24(726)	EPI_ISL_12845657	ON384030	G142D, L244S, L452R, T478K, T572I	21J (Delta)	AY.95
BSMMU_Viro_25(301)	EPI_ISL_12845658	ON384031	G142D, L452R, T478K, A522P	21A (Delta)	B.1.617.2
BSMMU_Viro_26(624)	EPI_ISL_7192041	ON384032	T19R, G142D, L452R, T478K, P681R, T732A, D950N, D1118Y	21A (Delta)	B.1.617.2
BSMMU_Viro_27(659)	EPI_ISL_12845659	ON384033	T95I, G142D, L452R, T478K, L552F, D614G, P681R, D950N, V1104L	21J (Delta)	B.1.617.2
BSMMU_Viro_28(657)	EPI_ISL_12845660	ON384034	Q14L, T19R, G142D, Q173H, L452R, T478K, S494L, D614G, P681R, D950N, P1263L	21A (Delta)	B.1.617.2
BSMMU_Viro_29(221)	EPI_ISL_12845661	ON384035	T19R, G142D, L452R, T478K, D614G, P681R, D950N	21A (Delta)	B.1.617.2
BSMMU_Viro_30(83)	EPI_ISL_12845662	ON384036	T19R, T95I, G142D, L452R, T478K, D614G, P681R, D950N	21J (Delta)	AY.119
BSMMU_Viro_31(687)	EPI_ISL_12845663	ON384037	T19R, G142D, L452R, T478K, A570G, T604I, D614G, P681R, D950N	21A (Delta)	B.1.617.2
BSMMU_Viro_32(82)	EPI_ISL_12845664	ON384038	G142D, L452R, T478K, D614G, P681R, D950N, T1117I	21A (Delta)	B.1.617.2

The study was approved by the institutional review board (IRB) of Bangabandhu Sheikh Mujib Medical University (IRB approval number BSMMU/2021/5963). Written informed consent was obtained from the patients before data collection. All information obtained from the study participants was kept confidential.

### Data availability.

The partial spike CDSs and metadata for all 30 samples were submitted to the GISAID database (www.gisaid.org), where they can be accessed through the accession numbers EPI_ISL_12845636 to EPI_ISL_12845658, EPI_ISL_7192041, and EPI_ISL_12845659 to EPI_ISL_12845664 (Table 1). The sequences can be also accessed at the NCBI database (GenBank accession numbers ON384009 to ON384038) (Table 1). The list of primers used to amplify the spike gene and the thermal cycling profiles for conventional PCR and cycle sequencing reactions can be viewed at https://doi.org/10.6084/m9.figshare.19882897.
